# Deleting multiple lytic genes enhances biomass yield and production of recombinant proteins by *Bacillus subtilis*

**DOI:** 10.1186/s12934-014-0129-9

**Published:** 2014-08-31

**Authors:** Yi Wang, Zhenmin Chen, Ruili Zhao, Tingting Jin, Xiaoming Zhang, Xiangdong Chen

**Affiliations:** State Key Laboratory of Virology, College of Life Sciences, Wuhan University, Wuhan, 430072 China; State Key Laboratory of Agricultural Microbiology, Huazhong Agricultural University, Wuhan, China; Hubei Provincial Cooperative Innovation Center of Industrial Fermentation, Wuhan, 430072 China

**Keywords:** *Bacillus subtilis*, Cell lysis, Biomass, Recombinant protein, Multiple gene silencing, Nattokinase

## Abstract

**Background:**

*Bacillus subtilis* is widely used in agriculture and industrial biotechnology; however, cell autolysis significantly decreases its yield in liquid cultures. Numerous factors mediate the lysis of *B. subtilis*, such as cannibalism factors, prophages, and peptidoglycan (PG) hydrolases. The aim of this work was to use molecular genetic techniques to develop a new strategy to prevent cell lysis and enhance biomass as well as the production of recombinant proteins.

**Results:**

Five genes or genetic elements representing three different functional categories were studied as follows: *lytC* encoding PG hydrolases, the prophage genes *xpf* and *yqxG-yqxH-cwlA* (*yGlA*), and *skfA* and *sdpC* that encode cannibalism factors. Cell lysis was reduced and biomass was enhanced by deleting individually *skfA*, *sdpC*, *xpf*, and *lytC*. We constructed the multiple deletion mutant LM2531 (*skfA sdpC lytC xpf*) and found that after 4 h of culture, its biomass yield was significantly increased compared with that of prototypical *B. subtilis* 168 (wild-type) strain and that 15% and 92% of the cells were lysed in cultures of LM2531 and wild-type, respectively. Moreover, two expression vectors were constructed for producing recombinant proteins (β-galactosidase and nattokinase) under the control of the P43 promoter. Cultures of LM2531 and wild-type transformants produced 13741 U/ml and 7991 U/ml of intracellular β-galactosidase, respectively (1.72-fold increase). Further, the level of secreted nattokinase produced by strain LM2531 increased by 2.6-fold compared with wild-type (5226 IU/ml vs. 2028 IU/ml, respectively).

**Conclusions:**

Our novel, systematic multigene deletion approach designed to inhibit cell lysis significantly increased the biomass yield and the production of recombinant proteins by *B. subtili*s. These findings show promise for guiding efforts to manipulate the genomes of other *B. subtilis* strains that are used for industrial purposes.

**Electronic supplementary material:**

The online version of this article (doi:10.1186/s12934-014-0129-9) contains supplementary material, which is available to authorized users.

## Background

*Bacillus subtilis* and related species such as *Bacillus licheniformis* and *Bacillus megaterium* are designated generally recognized as safe (GRAS) microorganisms [1]. Probiotic preparations of *B. subtilis* are commercially available worldwide as nutritional supplements and are used for preventing and treating gastrointestinal disorders such as diarrhea [2,3]. Further, *B. subtilis* is used to produce agents for treating plant and animal diseases, which may replace chemical germicides that are toxic to humans and other species and harm the environment [4-6]. Moreover, *B. subtilis* strains are major workhorses of industrial biotechnology and are widely employed as cell factories to produce useful enzymes such as proteases, amylases, and hydrolases as well as biochemicals such as riboflavin, nucleotides, nonribosomal peptides, antibiotics, and insecticides [7-9]. In particular, the ability of *B. subtilis* to secrete proteinaceous products into the growth medium greatly facilitates downstream processing, which is highly advantageous for industrial-scale fermentation [1].

Cell lysis occurs in liquid cultures of *B. subtilis*, which may significantly decrease the total biomass and reduce the fermentation yields. Therefore, we investigated whether inhibiting cell lysis increases biomass. *B. subtilis* genes involved in cell lysis are roughly divided into three categories [10-12]. The first category includes genes encoding products that play roles in cannibalism during sporulation. Nutrient limitation triggers the activation of Spo0A, the main regulator of sporulation, in a portion of cells. Spo0A regulates the *skf* and *sdp* operons. A small peptide encoded by *skfA*, the first gene of the *skf* operon, acts as an antibiotic that kills siblings. The products of *skfE* and *skfF* export the peptide antibiotic and confer resistance to it. Cells that do not express *spo0A* are killed, and their contents provide nutrients that feed their siblings and delay sporulation of the entire bacterial population. In cells that express Spo0A, the *sdp* operon produces the signaling protein SdpC that increases the killing effect [13-15].

Published studies mainly focus on the mechanism and biological significance of cannibalization; however, its effect on the biomass of liquid cultures of *B. subtilis* is unknown. Interestingly, the *skf* operon resides within prophage 1 [16], which may represent a defective viral genomic remnant. The second category of genes that influence the biomass of *B. subtilis* cultures includes prophages that can be activated and induce cell lysis. The *B. subtilis* genome harbors at least ten putative intact or partial prophages, and only PBSX and SPβ are lysogenic [10,17]. Heat shock treatment of the SP*βc2* mutant of *B. subtilis* 168 (wild-type) induces the production of high titers of SPβ [18]. However, this prophage is difficult to induce in wild-type *B. subtilis*, including the prototype strain 168, because the *c* gene encodes a strong repressor of SPβ induction [19,20]. The prophage PBSX exists in most of *B. subtilis* strain and it is induced by treating cells with agents that cause Survival-Oriented-Behavior responses, such as mitomycin C, hydrogen peroxide, UV irradiation, and thymidine starvation. Upon induction, random DNA fragments of approximately 13 kb derived from various sites of the host chromosome are packaged by PBSX proteins, and these defective phage particles are released by lysis of the host [21].

Induction of PBSX in normal bacterial cultures is inconspicuous, because only defective phage particles are released. Moreover, PBSX particles and subvirion components are detected in normal cultures of *B. subtilis* [22], suggesting that PBSX may be constitutively activated in a small percentage of cells during bacterial growth and consequently reduce cell density. The other eight prophages, including prophage 1, are considered chromosomal prophage remnants, because they lack the ability to replicate [17]. However, at least one PG hydrolase encoded by the lysis gene *skin* (*cw1A*), hydrolyzes the *B. subtilis* cell wall in vitro [23], indicating that these inactive prophages may influence the yield of bacterial biomass.

The third category of genes that mediate cell lysis encode PG hydrolases that are expressed during vegetative growth. PG (or murein) is a continuous covalent heteropolymer that extends to the outer side of the cytoplasmic membrane of most bacteria [11]. PG hydrolases mediate functions such as cell growth, cell separation, cell wall turnover, cell mobility, sporulation, and germination [10] and include the vegetative cell wall hydrolases LytC (CwlB), LytD (CwlG) [24], LytE (CwlF) [25], LytF (CwlE) [26], LytG (YubE) [27], CwlO (YvcE) [28], CwlS (YojL) [29] and CwlK (YcdD) [30]. Only LytC, LytD, LytG, and LytE have been proven to mediate cell lysis [10,27]. LytC and LytD possess amidase and *N*-acetylglucosaminidase activities, respectively, and transcription of the both genes is controlled by the sigma factor σ^D^. LytC is considered as the critical PG hydrolase that mediates autolysis of vegetative cells, because individual deletion of *lytC* can effectively inhibit cell lysis [24,31]. LytD seems to influence cell lysis when accompanying with LytC, since disruption of only *lytD* does not significantly affect sodium azide-induced cell lysis, but a *lytC lytD* double mutant exhibits less cell lysis than a *lytC* single mutant [24]. Similar to *lytD*, *lytG* encodes another *N*-acetylglucosaminidase, which is also a minor PG hydrolases [27]. Meanwhile, another lysis gene *lytE* encodes an endopeptidase. Single mutant of *lytE* displayed a growth defect and cell lysis reduction. However, LytE had no significant effect on the degradation of isolated cell wall preparations and was predicted to facilitate the action of LytC [25]. Further, a *lytE cwlO* double mutant undergoes synthetic lethality for the defect in lateral cell wall metabolism and cell elongation [32,33].

Here, we describe the development of a molecular genetics approach designed to delete multiple genes involved in cell lysis to improve biomass and the production of recombinant proteins. The results show that individually deleting *skfA*, *sdpC*, *xpf* [34], or *lytC* decreased cell lysis and increased biomass compared with wild-type. Deleting different combinations of these genes further increased biomass, and a quadruple mutant produced a high level of biomass and least cell lysis. Moreover, higher levels of β-galactosidase and nattokinase were produced by the quadruple mutant. In contrast, there was no detectable effect on β-galactosidase or nattokinase production by a mutation of *yGlA*, the *yqxG-yqxH-cwlA* gene cluster related to the hydrolase of phage *skin*. These findings may be relevant for manipulating other industrial *Bacillus* strains that ubiquitously harbor homologous genes.

## Results

### Analysis of biomass of *B. subtilis* strains harboring mutations in individual genes encoding proteins involved in cell lysis

**Figure 1:**
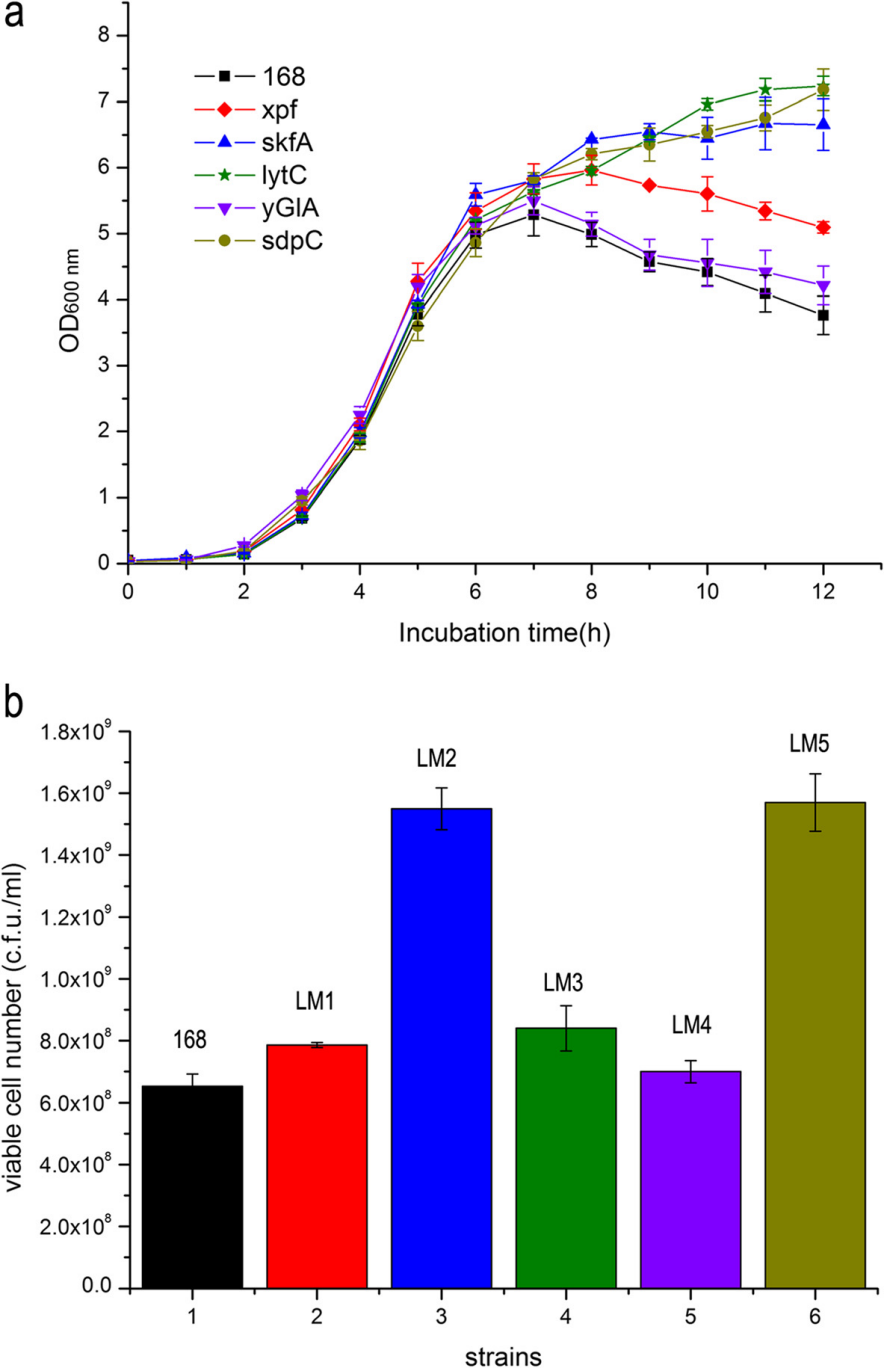
**Biomass yields of single lytic-gene deletion mutants. (a)** OD_600_ of cultures of wild-type and single-deletion mutants: ■ *B. subtilis* 168 (Wild-type), ◆ LM1, ▲LM2, ★ LM3, ▼ LM4, ●LM5. **(b)** 12-h viable cell numbers: 1 Wild-type, 2 LM1, 3 LM2, 4 LM3, 5 LM4, 6 LM5. The data presented represent the mean and standard deviation (SD) of triplicate measurements of three different colonies of the same genotype for each mutant.

To test the influence of targeted mutation of genes involved in cell lysis on the biomass of batch cultures of *B. subtilis*, we generated the deletion mutants LM1 (*xpf*), LM2 (*skfA*), LM3 (*lytC*), LM4 (*yGlA*), and LM5 (*sdpC*), respectively (Additional file 1: Table S1, Appendix S1). The mutants and wild-type were inoculated into LB medium and cultured in 250-mL flasks at 37°C with shaking for 12 h. Consistent with published data [35], the transition of wild-type from exponential growth to stationary phase occurred between 6 to 7 h, after which the optical density measured at 600 nm (OD_600_) decreased, indicating autolysis (Figure 1a). The growth rates of the wild-type and five mutants were not significantly different before 7 h. However, there was a noticeable difference among these strains immediately after they entered stationary phase (7–12 h). For example, the OD_600_ values of cultures of strains LM2 (*skfA*), LM3 (*lytC*), and LM5 (*sdpC*) continued to increase after 7 h and were approximately twice that of wild-type at 12 h. The OD_600_ of the *xpf* mutant culture was higher than that of wild-type after 12 h. Only the growth of LM4 (*yGlA*) (OD_600_ = 4.2) was similar to that of the wild-type (OD_600_ = 3.7) at 12 h, indicating that the PG hydrolase encoded by *skin* may not contribute to cell lysis.

Consistent with the OD_600_ data, the number of viable cells (colony forming units) in cultures of LM2 (*skfA*) and LM5 (*sdpC*) were twice that of wild-type (Figure 1b). The cell viability data for LM1 (*xpf*) and LM4 (*yGlA*) were consistent with the corresponding OD_600_ values and were in agreement as well with those of the wild-type strain. In contrast, the high OD_600_ value of the LM3 (*lytC*) culture was not consistent with its viable count at 12 h (Figure 1a), which was only slightly higher than that of the parental strain (Figure 1b). To resolve this discrepancy, we observed cell morphology and found that mutants LM1 (*xpf*), LM2 (*skfA*), LM5 (*sdpC*), and LM4 (*yGlA*) appeared similar to the parental strain; however, LM3 (*lytC*) formed numerous chains (Additional file 1: Figure S2). Therefore, although the biomass of the *lytC* mutant increased compared with the parental strain, the formation of chains led to an underestimation of the number of viable cells. These results demonstrate that deleting *skfA*, *lytC*, *sdpC*, or *xpf* significantly increased biomass compared with wild-type, although deletion of *yGlA* had little effect.

### Analysis of biomass of *B. subtilis* strains harboring mutations in multiple genes encoding proteins involved in cell lysis

We next generated six double-knockout strains as follows: LM12 (*xpf skfA*), LM13 (*xpf lytC*), LM15 (*xpf sdpC*), LM23 (*skfA lytC*), LM25 (*skfA sdpC*), and LM35 (*lytC sdpC*)*.* The biomass of each of the double mutants increased compared with that of the cognate single mutant after culture at 37°C for 12 h (Additional file 1: Figure S3a). However, the influence of these double mutations was difficult to deduce from the performance of the respective single mutant. For example, we predicted that *xpf* would contribute the least to biomass compared with the other three strains; however, the OD_600_ of the LM15 (*xpf sdpC*) culture was the highest and exceeded that of LM23 (*skfA lytC*) by 14%. Moreover, the numbers of viable cells of the double mutants were higher than that of their cognate single mutants, but not in complete agreement with the OD values. For example, although the OD_600_ of the LM25 (*skfA sdpC*) culture was not the highest, its viability was the highest and exceeded that of the parental strain by a factor of approximately 3 (Additional file 1: Figure S3b). Moreover, the viabilities of cultures of LM35 (*lytC sdpC*) and LM23 (*skfA lytC*) were lower when *lytC* was deleted compared with those of the other double mutants (Additional file 1: Figure S3b).

**Figure 2:**
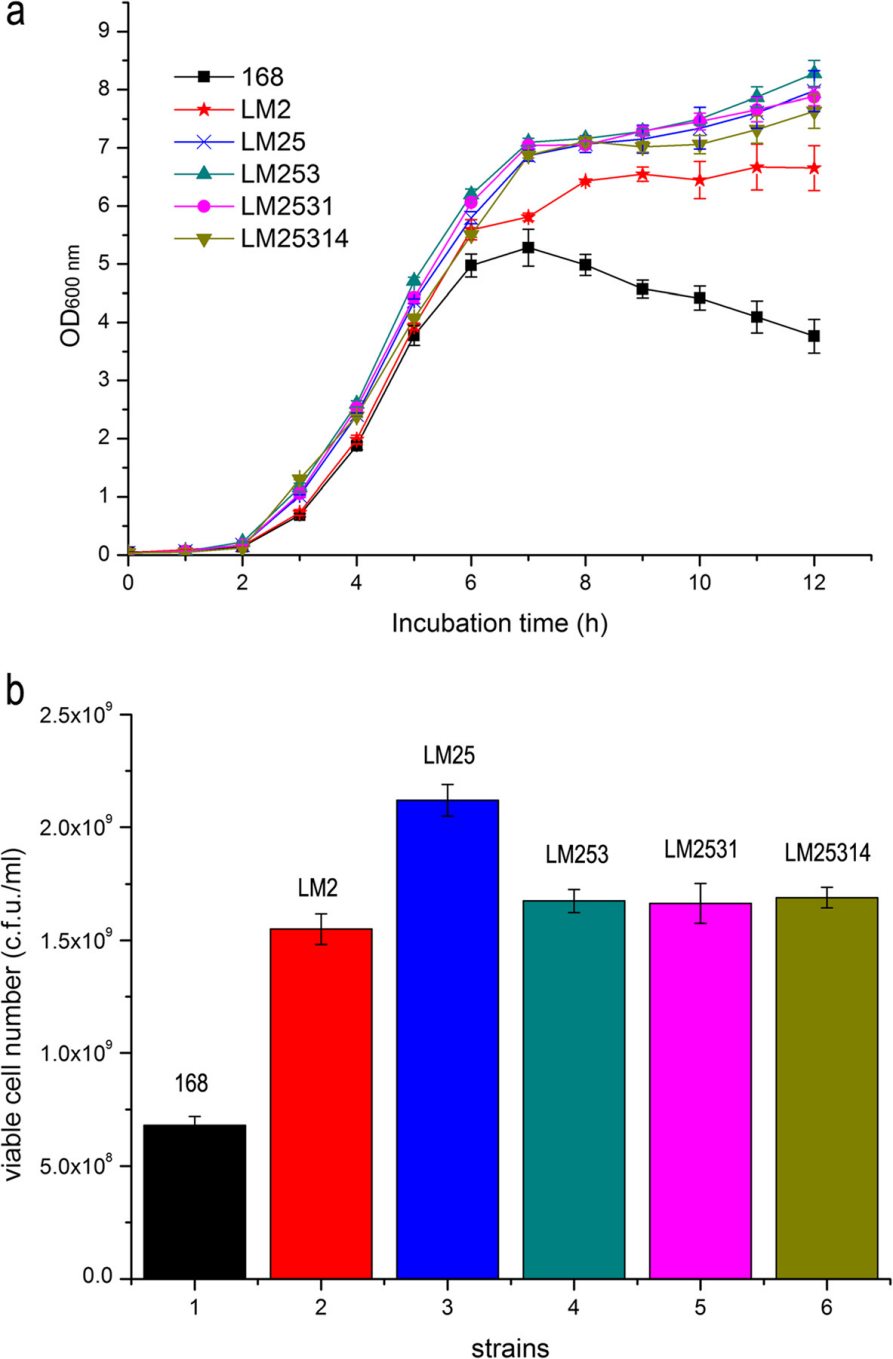
**Biomass yields of multiple lytic-gene deletion mutants. (a)** OD_600_ of cultures of wild-type and multiple-deletion mutants:■ Wild-type, ★ LM2, × LM25, ▲ LM253, ● LM2531, ▼ LM25314; **(b)** 12 h viable cell numbers: 1 *B. subtilis* 168, 2 LM2, 3 LM25, 4 LM253, 5 LM2531, 6 LM25314. There was no significant difference between the biomass of each of the two sets of multiple-deletion mutants, and therefore data for one set is presented here. The data represent the mean and SD from triplicate measurements of three different colonies of the same genotype for each mutant.

We next constructed two sets of mutants with multiple deletions in the order as follows: *skfA*, *sdpC*, *lytC*, *xpf*, *yGlA* and *lytC*, *sdpC*, *xpf*, *skfA*, *yGlA*. The growth curves and numbers of viable cells of the first series of mutants are shown in Figure 2. Although the OD_600_ value of the triple mutant (LM253) culture after 12 h was slightly higher than that of the double mutant (LM25), mutating more than two genes did not significantly increase biomass. The formation of chains by *lytC* deletion mutants once again caused an underestimation of the viable cell count based on the OD_600_ values (Additional file 1: Figure S4).

### Autolytic activity of deletion mutants

**Figure 3:**
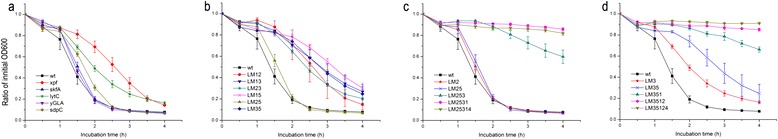
**Effect of sodium azide on lysis of exponential-phase cultures. (a)** Lysis of single mutants: ■ wild-type, ◆ LM1, ▲LM2, ★ LM3, ▼ LM4, ●LM5. **(b)** Lysis of double mutants: ■ wild-type, ●LM12, ▼ LM13, ★ LM23, × LM15, ▲ LM25, ◆ LM35. **(c)** First set of multiple mutants: ■ wild-type, ★ LM2, × LM25, ▲ LM253, ● LM2531, ▼ LM25314. **(d)** Second set of multiple mutants: ■ wild-type, ★ LM3, × LM35, ▲ LM351, ● LM3512, ▼ LM35124. The data represent the mean and SD from triplicate measurements of three different colonies of the same genotype for each mutant.

Sodium azide is a cytochrome oxidase inhibitor that inhibits cell growth and it is used to assess the tendency of a cell to lyse under certain conditions [10]. When the *B. subtilis* strains were cultured with shaking in LB medium at 37°C for 3.5 h (mid-log phase), sodium azide was added to a final concentration of 0.05 M and cell lysis was monitored by measuring OD_600_. Wild-type cells started to lyse immediately after sodium azide was added, and only 9% and 8% cells were intact after 3 and 4 h, respectively. The results were similar for LM2 (*skfA*), LM5 (*sdpC*), and LM4 (*yGlA*). In contrast, 25% and 15% of the cells in the LM3 (*lytC*) culture were intact after 3 and 4 h, respectively, in agreement with the results of previous studies [10,31]. Cell lysis was further reduced in cultures of LM1 (*xpf*) (35% and 14% of intact cells after 3 and 4 h, respectively) (Figure 3a).

There were fewer lysed cells in cultures of the double mutants with deletions of *xpf* or *lytC*, namely LM12 (*xpf skfA*), LM13 (*xpf lytC*), LM15 (*xpf sdpC*), LM23 (*skfA lytC*), and LM35 (*lytC sdpC*) in contrast to cultures of LM25 (*skfA sdpC*), which lysed to a similar extend compared with wild-type (Figure 3b).

Although the extents of cell lysis in cultures of the single mutant LM2 (*skfA*) and double mutant LM25 (*skfA sdpC*) were similar compared with wild-type, only 40% the cells in cultures of the triple mutant LM253 (*skfA sdpC lytC*) were lysed after 4 h (Figure 3c). Further, only 15% and 18% of the cells lysed after 4 h in cultures of the quadruple mutant LM2531 (*skfA sdpC lytC xpf*) and quintuple mutant LM25314 (*skfA sdpC lytC xpf yGlA*). These observations indicate that deletion of *xpf* and *lytC* exerted a significant effect on cell lysis.

The effects of the *skfA* and *sdpC* mutations were significant as well. Although the extent of lysis in cultures of either of the single mutants LM2 (*skfA*) or LM5 (*sdpC*) or the double mutant LM25 (*skfA sdpC*) was not significantly altered, only 15% of the cells in cultures of the quadruple-mutant LM2531 (*skfA sdpC lytC xpf*) were lysed after 4 h compared with 72% lysis in cultures of the double-mutant LM13 (*xpf lytC*) (Figure 3c). Moreover, cell lysis decreased when we deleted genes sequentially in the order *lytC*, *sdpC*, *xpf*, *skfA*, and *yGlA* as follows: wild-type, 92%; single-mutant LM3 (*lytC*), 84%; double-mutant LM35 (*lytC sdpC*), 75%; triple-mutant LM351 (*lytC sdpC xpf*), 34%; quadruple-mutant LM3512 (*lytC sdpC xpf skfA*), 15%; and quintuple-mutant LM25314 (*lytC sdpC xpf skfA yGlA*), 10%. These findings indicate that *skfA* or *sdpC* exerted a combined influence on cell lysis when *xpf* or *lytC* were mutated.

Therefore, we recommend a combined deletion of *lytC*, *sdpC*, *xpf*, and *skfA* for constructing *B. subtilis* cell factories. Although the biomass of cultures of the quadruple mutant was similar to those of the double or triple mutants (Figure 2), it exhibited increased resistance to lysis under adverse conditions (e.g. sodium azide) (Figure 3c and d). Deletion of *yGlA* was the only mutation that did not detectably influence cell lysis (Figure 3) or biomass (Figures 1, 2).

### Production of recombinant proteins by the quadruple deletion mutant

**Figure 4:**
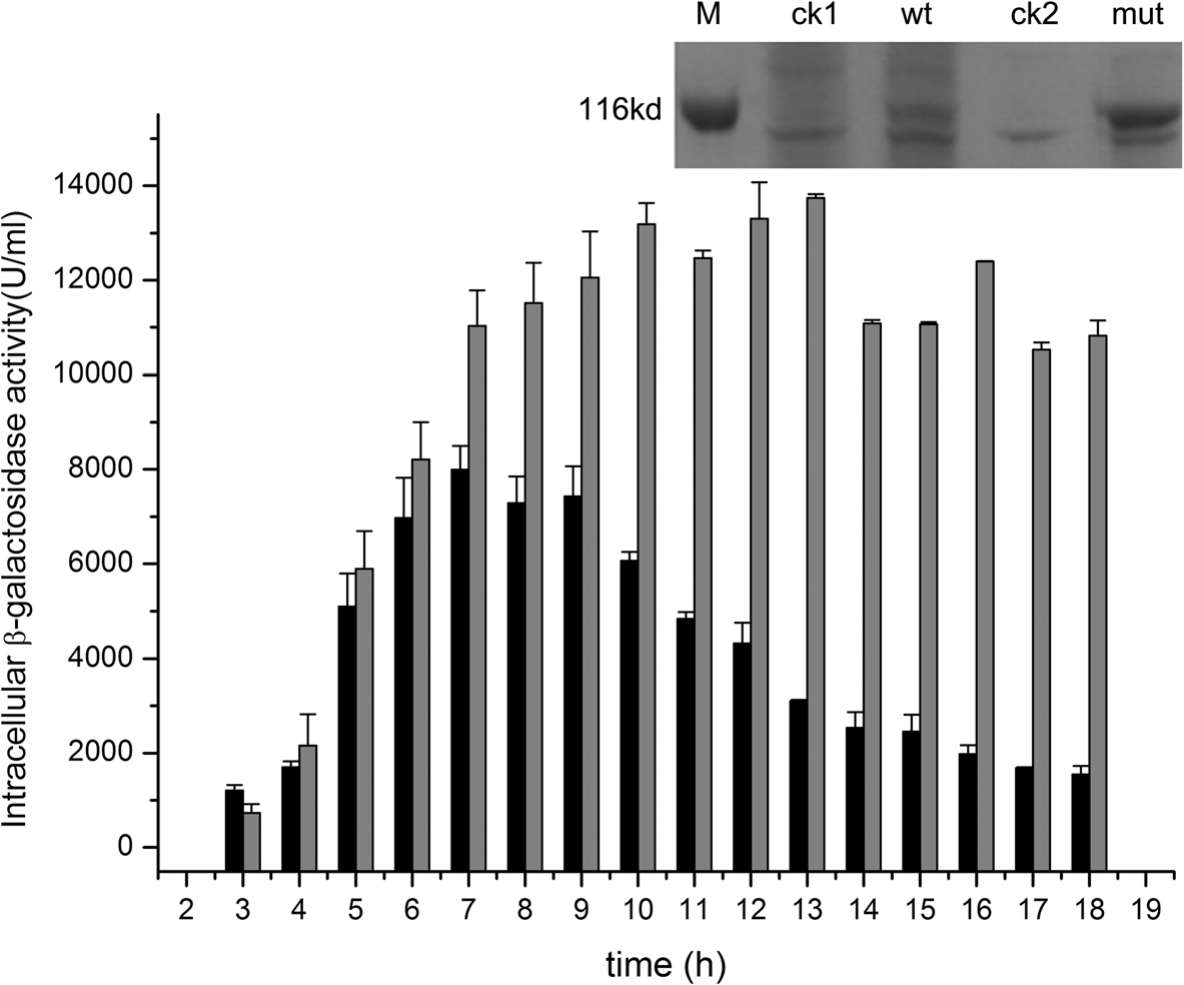
**Intracellular production of β-galactosidase.** Wild-type (wt) (black columns), LM2531 (mut) (gray columns). Inset: SDS-PAGE analysis of cell pellets. Standard (M), *B. subtilis* 168/pBE2 (ck1), *B. subtilis* 168/pBL (wt), LM2531/pBE2 (ck2), LM2531/pBL (mut).

We next tested the ability of the quadruple mutant to express recombinant β-galactosidase and nattokinase by using the expression vectors pBL and pBNA, respectively (Additional file 1: Appendix S1, Figure S1). There were similar levels of β-galactosidase in cell pellets of LM2531 and the wild-type during exponential growth (<7 h); however, they differed significantly upon entry into stationary phase, consistent with their biomass yields. The β-galactosidase activity of the wild-type peaked at 7 h and then declined, in contrast, to that of LM2531, which continued to increase after 7 h, peaked at 13 h at a level 1.72 times higher than that of wild-type, and remained constant thereafter (at least until 18 h). The results of SDS-PAGE analysis of β-galactosidase expression by cells harvested after 12 h of culture are consistent with the activity data (Figure 4). Note that, although the β-galactosidase encoded by pBL lacked a secretory signal sequence and therefore expressed intracellularly (Additional file 1: Appendix S1, Figure S1), β-galactosidase activity was detected in the supernatants of cultures of both strains, particularly in stationary phase, likely due to cell lysis. However, β-galactosidase activity in the supernatant of 168/pBL was significantly higher than that of LM2531/pBL at 12 h, indicating significant inhibition of cell lysis of the quadruple-deletion mutant.

**Figure 5:**
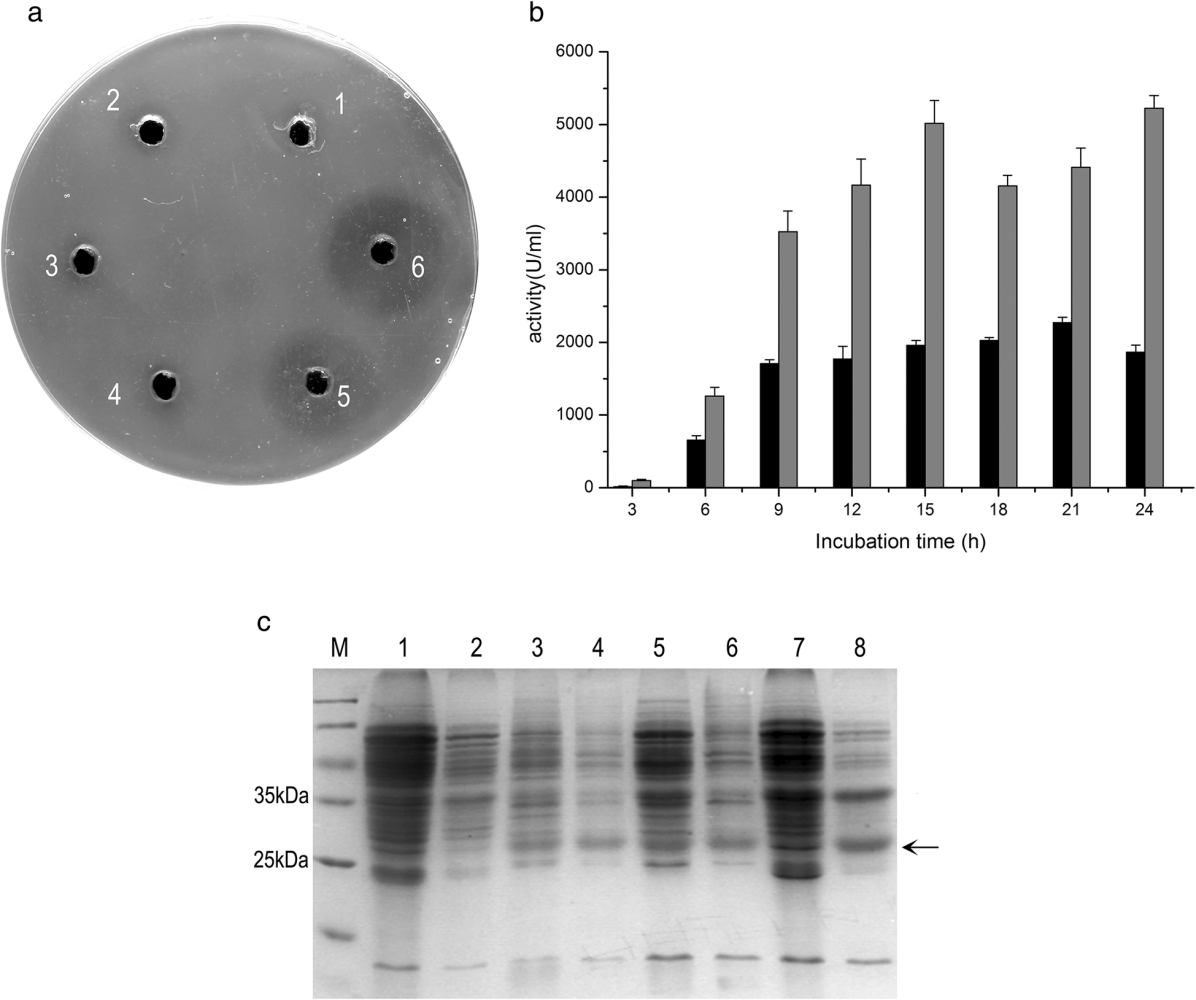
**Nattokinase production in supernatants of wild-type and mutant cultures. (a)** Nattokinase activity in the supernatants of wild-type and mutant cultures, Wild-type (wt) (black columns), LM2531 (mut) (gray columns). **(b)** SDS-PAGE analysis. 1. *B. subtilis* 168/pBE2 supernatant, 2. LM2531/pBE2 supernatant as control, 3. *B. subtilis* 168/pBNA supernatant at 6 h, 4. LM2531/pBNA supernatant at 6 h, 5. *B. subtilis* 168/pBNA supernatant at 12 h, 6. LM2531/pBNA supernatant at 24 h, 7. *B. subtilis* 168/pBNA supernatant at 24 h, 8. LM2531/pBNA supernatant at 24 h. The arrow indicates the position corresponding to mature nattokinase (29 kDa). **(c)**: fibrinolytic activity assay. 1. *B. subtilis* 168/pBE2 supernatant, 2. LM2531/pBE2 supernatant, 3. *B. subtilis* 168/pBNA cell lysate, 4. LM2531/pBNA cell lysate, 5. *B. subtilis* 168/pBNA supernatant, 6. LM2531/pBNA supernatant.

Because the ability to secrete recombinant proteins is a major advantage of using *B. subtilis*, we transformed the quadruple-mutant LM2531 with a plasmid (pBNA) expressing nattokinase containing a secretory signal peptide. The supernatants and cells harvested from cultures of LM2531 and wild-type, each transformed with pBNA, were assayed for fibrinolysis catalyzed by nattokinase (Figure 5a). Fibrinolytic activity was detected only in supernatants of both cultures and was higher by a factor of 2.6 in supernatants of LM2531 cultures (Figure 5b). The expression of nattokinase in culture supernatants harvested at 6 (log phase), 12 (stationary phase), and 24 h (death phase) was analyzed using SDS-PAGE (Figure 5c). A major band of approximately 29 kDa corresponding to mature nattokinase [36] was detected at each sampling time (lanes 3–8) but not in cultures of untransformed cells (lanes 1 and 2). The amounts of nattokinase in LM2531 supernatants (lanes 4, 6, 8) were greater compared with those of the wild-type (lanes 3, 5, 7). Moreover, the total amount of wild-type proteins in the supernatant increased from 6 to 24 h (lanes 4, 6, 8), indicating increased cell lysis compared with mutant cultures.

## Discussion

Lysis of *B. subtilis* strains used for industrial purposes significantly decreases product yield [7,37]. We addressed this issue here by systematically deleting genes involved in autolysis from the wild-type strain *B. subtilis* 168 and assessed the effects of single and multiple deletions on cell lysis and biomass production.

SkfA and SdpC are sibling-killing factors that are implicated in the sporulation of *B. subtilis* [15,16]; however, their role in other growth phases is unknown. We show here that *skfA* and *sdpC* were transcribed during log phase (Additional file 1: Figure S5), which is consistent with our findings that cell lysis was simultaneously attenuated (Figure 3). However, the effect of deleting *skfA* and *sdpC* was only detected when *xpf* or *lytC* were also deleted, suggesting that their expression levels were relatively low and therefore undetectable when *xpf* and *lytC* are expressed by the wild-type. We reasoned that the effects of lytic enzymes might be redundant. Therefore, we constructed mutants with multiple deletions of lytic genes. The highest numbers of intact cells were detected in cultures of the quadruple-mutant LM3512 (*lytC sdpC xpf skfA*). However, deleting *skfA* or *sdpC* from LM13 (*xpf lytC*) did not cause a further increase in biomass (Figure 2), suggesting that the balance between cell lysis and cell growth is very complex and requires further investigation.

Lysis of *B. subtilis* is mediated by the products of prophage genes. Although the defective prophage PBSX does not influence the growth, proliferation, or sporulation of *B. subtilis* [38,39] we show here that the sigma-like prophage PBSX factor Xpf is involved in the normal growth and differentiation of *B. subtilis*. Although *xpf* was deleted from the LM1 mutant, cell lysis was effectively decreased, it produced more spores than wild-type (Additional file 1: Figure S6A). To the best of our knowledge, this is the first evidence that links this defective prophage to cell lysis and biomass accumulation in *Bacillus*. The prophage *skin* element harbors phage-like operon sequences that are highly homologous to segments of the regulatory region and late operon of PBSX, but there are 17 more ORFs with the region of *skin* since significant rearrangements. Unlike PBSX, *skin* cannot be induced by SOS response [40]. Our present results confirm speculation that this prophage is not active in *B. subtilis* [40], because all the mutations of *yGlA* exhibited no obvious effects on biomass yield or cell autolysis.

PG hydrolases represent a distinct category of lytic enzymes. LytC is the major vegetative PG hydrolase expressed by *B. subtilis*, and it mediates cell separation, cell wall turnover, antibiotic-induced lysis, and motility [24]. Here we show that the deletion mutant of *lytC* (LM3) was more resistant to cell lysis compared with wild-type and formed chains, in contrast to the wild-type and other mutant strains (Additional file 1: Figure S2).

Because inactivating PG hydrolases is not lethal, it was possible to construct deletion mutants. PG hydrolases such as LytD, LytE, and LytG disrupt the cell wall in vitro [24,25,27]. However, we did not delete them all, because lack of PG hydrolase activity may interfere with the ability of cells to separate or decrease membrane permeability, which decreases protein yields [11]. Therefore, a balance between resistance to lysis and efficient protein expression is required. The mutant LM2531 performed well in this regard.

Although there are published studies on the relationship between certain lytic genes and biomass production, none addresses the combined effects of the products of these genes on cell lysis and biomass yield. We demonstrate here that cultures of the quadruple mutant LM2531 exhibited the least cell lysis and high biomass yield compared with wild-type. Moreover, LM2531 expressed significant higher levels of intracellular recombinant β-galactosidase for a prolonged period and secreted recombinant nattokinase compared with the wild type strain.

We believe that the enhanced resistance of LM2531 to cell lysis and its ability to efficiently express recombinant proteins may be attributed to its production of increased numbers of cells. Although the amount of protein correlates with cell number, there are exceptions. For example, individual silencing of *lytC* and *SpoIIGA* in the *Bacillus* strain ATCC 6051 increased cell densities; however, only the *lytC* mutant produced increased levels of alpha-amylase [41]. SpoIIGA is a membrane-associated aspartic protease responsible for activating the early sporulation sigma factor E [42]. Therefore, we reasoned that the production of recombinant protein might be optimized before *spoIIGA* is transcribed. Thus, vegetative genes may be a better choice for this purpose. This assumption is supported by the outcome of deleting four genes from the wild-type that are expressed during the vegetative phase.

A second explanation to account for the phenotype of LM2531 is the deletion of genes that prevent sporulation. Thus, after 12 h, approximately 50% of the wild-type cells formed spores, in contrast to less than 10% in cultures of the quadruple mutant (Additional file 1: Figure S6B). The presence of spores reduces the yield of target products and increase production costs, because it is difficult to remove spores from bioreactors [43]. Therefore, inhibiting spore formation will likely benefit the production of recombinant proteins.

Third, the inactivation of lysis genes inhibited leakage of cellular contents during cell lysis. For example, the inactivation of *lytC* prevents the leakage of intracellular proteins into the culture fluid [31]. Consistent with these results, we show here that the amount of total extracellular proteins was high in wild-type cultures. In contrast, they were present at extremely low levels in LM2531 cultures (Figure 5c). Cell lysis releases proteases into the culture fluid that may degrade secreted recombinant proteins. Therefore, inhibiting cell lysis may lead to higher yields of recombinant protein produced by the mutant. Moreover, minimizing the amount of degradation products and materials released by lysed cells will simplify downstream purification processes.

## Conclusions

Here we implemented a new strategy to enhance biomass yield and prevent cell autolysis in liquid cultures of wild-type *B. subtilis* 168 by creating mutants with deletions of multiple lytic genes. The production of intracellular and secreted recombinant proteins was significantly elevated in cultures of the mutant LM2531 by the deletion of *lyt*C, which encodes a major PG hydrolase; prophage-encoded *xpf*; and cannibalism factors *skfA* and *sdpC*. Because these lytic genes are conserved among *Bacillus* species, the results of our experiments performed here using *B. subtilis* 168 will provide a guideline for genetic manipulations of other *Bacillus* strains to enhance the production of industrial materials.

## Methods

### Strains and plasmids

All strains and plasmids used in this study are listed in Additional file 1: Tables S1 and Additional file 1: Table S2. *B. subtilis* mutants were derived from wild-type *B. subtilis* 168 (CCTCC AB93017). *Escherichia coli* DH5α was used as the host for plasmid construction. The *E. coli*/*B. subtilis* shuttle vector pNNB194 [44] was used to generate gene deletions and replacements. Plasmid pBE2 was used to construct vectors for expressing recombinant proteins [45]. Genetic procedures for constructing plasmids and mutants are shown in Additional file 1: Appendix S1.

### Measurement of biomass and cell viability

The biomass of *B. subtilis* cultures was evaluated according to optical density (OD) and viable cell number. Overnight cultures of wild-type and mutants were inoculated into LB medium at a dilution of 1:100 and shaken on a reciprocating shaker at 200 rpm at 37°C for 12 h. The OD at 600 nm was measured at 1 h intervals, and serial dilutions of 12-h cultures were plated onto LB agar. The numbers of colonies were counted after incubation for 16 h. All the experiments were performed in triplicate using three different colonies of the same genotype.

### Analysis of spore formation

The number of spores was evaluated by colony counts after heat-shock treatment of 80°C for 20 min followed by immediate cooling in water to room temperature. Serially diluted samples (100 μl) were spread on LB plates. After the plates were incubated at 37°C for 16 h, the number of colonies was used to calculate the number of spores in the sample. All the experiments were performed in triplicate using three different colonies of the same genotype.

### Measurement of cell autolysis

Wild-type and mutants were grown to mid-exponential phase (3.5 h) in LB medium. After adding sodium azide to a final concentration 0.05 M (MIC × 10), the OD_600_ of the cultures was measured at 30-min intervals at 37°C and 200 rpm. The ratio of OD_600_ at each sampling time to the initial OD_600_ was used to calculate cell lysis [10,46]. All the experiments were performed in triplicate using three different colonies of the same genotype.

### β-Galactosidase assays

Recombinant *B. subtilis* strains carrying pBL were grown in LB medium supplemented with 10 μg/ml of kanamycin. Samples (0.2 ml) were taken at 1 h intervals and centrifuged. The pellets were resuspended in 1 ml of Z buffer [47] containing 0.1 mg/ml of lysozyme, and incubated at 37°C for 20 min to remove cell walls. The cells were permeabilized by adding 0.1% Triton X-100, one drop of 0.1% SDS, and two drops of chloroform. After equilibrating the tubes in a 30°C water bath for 5 min, 0.2 ml ONPG was added to each tube. After incubation at 30°C for 2 min, the reaction was stopped by adding 0.5 ml of 1 M Na_2_CO_3_. Absorbance was recorded at 420 nm using a spectrophotometer. β-Galactosidase activity was defined as the amount of enzyme required to release 1 nmol of 2-nitrophenol from ONPG per min in 1 ml of culture fluid. The equation to calculate β-galactosidase activity [47,48] is as follows:

$$ \mathrm{Activity}=\frac{\mathrm{OD}420}{\mathrm{t}\times \mathrm{vol}}\times \frac{1\ \mathrm{nmol}}{0.0045\ \mathrm{ml}\ \mathrm{cm}}\times 1.7\ \mathrm{ml} $$

Where t min = reaction time (2 min)

vol ml = volume of culture

0.0045 OD_420_/nmol = extinction coefficient of 2-nitrophenol

1.7 ml = total volume of reaction mixture

cuvette = 1 cm path length

Activity = nmol/min/ml = U/ml

Cell pellets (approximately 100 μl) were disrupted using an ultrasonicator and were analyzed using SDS-PAGE.

### Fibrinolytic enzyme assays

Nattokinase was first identified in the Japanese fermented food *natto*. It is widely used to preventing thrombotic diseases because it is a food and exerts strong fibrinolytic activity [49]. We PCR-amplified the gene encoding nattokinase from *B. natto* strain BN13. The amplicon contained an open reading frame (ORF) of 1143 bp starting with GTG that was predicted to encode a 29-residue secretory signal peptide, a 77-residue propeptide, and a 275-residue mature enzyme (approximately 29 kDa) [36]. The entire ORF was used to construct plasmid pBNA (For details, Additional file 1: Appendix S1).

*B. subtilis* strains transformed with pBNA were grown in LB medium supplemented with 10 μg/ml of kanamycin. Samples were taken at 3-h intervals. After centrifugation, cell pellets and supernatants were collected. The pellets were washed twice with phosphate-buffered saline (PBS), resuspended into the same volume of PBS buffer, and disrupted using an ultrasonicator. Fibrinolytic activity was determined by measuring the areas of the clear zone on the fibrin plates, which were prepared according to Astrup and Sterndorff [50]. The supernatant or disrupted cell suspension (10 μl each) was added to each hole. Using commercial urokinase as a standard, we generated a standard curve (see Additional file 1: Figure S7) to determine fibrinolytic activity. Approximately 500 μl of supernatant or 100 μl of sonicated cell pellets was analyzed using SDS-PAGE.

## Additional file

Additional file 1: Table S1.
*B. subtilis* and *E. coli* strains. **Table S2.** Plasmids. **Table S3.** Primers. **Appendix S1.** Molecular genetic procedures for constructing plasmids and mutants. **Figure S1.** Construction of expression plasmid. **Figure S2.** Characteristic morphologies of the single-deletion mutants. **Figure S3.** Biomass yields of double lytic-gene deletion mutants. **Figure S4.** Characteristic morphologies of the multiple-deletion mutants. **Figure S5.** RT-PCR analysis of *skfA* and *sdpC* transcription in *B. subtilis*. **Figure S6.** Spore production by mutant strains. **Figure S7.** Nattokinase standard curve.

## Abbreviations

*yGlA*: Gene cluster *yqxG-yqxH-cwlA*
PG: Peptidoglycan
