# Supplementary material for: Deleting multiple lytic genes enhances biomass yield and production of recombinant proteins by Bacillus subtilis
Source: Microb Cell Fact. 2014 Aug 31;13:129. doi: 10.1186/s12934-014-0129-9 (PMC4243946; doi:10.1186/s12934-014-0129-9)
Supplement: Additional file 1: Table S1. — B. subtilis and E. coli strains. Table S2. Plasmids. Table S3. Primers. Appendix S1. Molecular genetic procedures for constructing plasmids and mutants. Figure S1. Construction of expression plasmid. Figure S2. Characteristic morphologies of the single-deletion mutants. Figure S3. Biomass yields of double lytic-gene deletion mutants. Figure S4. Characteristic morphologies of the multiple-deletion mutants. Figure S5. RT-PCR analysis of skfA and sdpC transcription in B. subtilis. Figure S6. Spore production by mutant strains. Figure S7. Nattokinase standard curve. [file 12934_2014_129_MOESM1_ESM.doc]

**Table S1 *B. subtilis* and *E.coli* strains**

| Strains | Genotype and/or relevant characteristic(s) | Source(s) and/or reference |
| --- | --- | --- |
| *B. subtilis* strains | | |
| 168 | Lab strain, trp^-^ ile^-^ ura^-^, Erm^s^ | CCTCC AB93017 |
| LM1 | *xpf* | This work |
| LM2 | *skfA* | This work |
| LM3 | *lytC* | This work |
| LM4 | *yqxG-ygxH-cwlA* (named as *yGlA* fragment) | This work |
| LM5 | *sdpC* | This work |
| LM12 | *xpf skfA* | This work |
| LM13 | *xpf lytC* | This work |
| LM15 | *xpf sdpC* | This work |
| LM23 | *skfA lytC* | This work |
| LM25 | *skfA sdpC* | This work |
| LM253 | *skfA sdpC lytC* | This work |
| LM2531 | *skfA sdpC lytC xpf* | This work |
| LM25314 | *skfA sdpC lytC xpf yGlA* | This work |
| LM35 | *lytC sdpC* | This work |
| LM351 | *lytC sdpC xpf* | This work |
| LM3512 | *lytC sdpC xpf skfA,* genetically same to LM2531 | This work |
| LM35124* | *lytC sdpC xpf skfA yGlA,*  genetically same to LM25314 | This work |
| BN13 | *Bacillus natto* produce nattokinase | Our lab |
| *E.coli* strains |  |  |
| DH5α | *supE*44 ∆*lacU*169(φ80*lacZ*∆*M*15) *hsdR*17 *recA*1 *endA*1 *gyrA*96 *thi*-1 *relA*1 | Our lab |
| MG1655 | F^-^ *λ^-^ ilvG rfb-50 rph-1* | Our lab |

* LM is short for lysis gene mutant. 3 for *lytC*,5 for *sdpC*,1for *xpf*,2 for *skfA*,4 for *yGlA*, and their order corresponds to the order in which they were knockout.

**Table S2 Plasmids**

| Plasmids | Genotype and/or  relevant characteristic(s) | Source(s)and/or  reference |
| --- | --- | --- |
| pUC18 | Cloning vector, Amp^r^ | Our lab |
| Integrative vectors |  |  |
| pNNB194 | *B.subtilis-E.coli* shuttle vector, hybrid between pE194 and pBluesript II SK+, Erm^r^ Amp^r^ | Connelly et al., 2004 |
| pNNB194-Δ*xpf* | *xpf* deletion vector, Erm^r^ Amp^r^ | This work |
| pNNB194-Δ*skfA* | *skfA* deletion vector, Erm^r^ Amp^r^ | This work |
| pNNB194-Δ*lytC* | *lytC* deletion vector, Erm^r^ Amp^r^ | This work |
| pNNB194-Δ*sdpC* | *sdpC* deletion vector, Erm^r^ Amp^r^ | This work |
| pNNB194-Δ*yGlA* | *yqxG-ygxH-cwlA*( named as *yGlA*)  deletion vector, Erm^r^ Amp^r^ | This work |
| Expression vectors |  |  |
| pBE2 | *B.subtilis-E.coli* shuttle vector, hybrid between pUB110 and pBR322, Kan^r^ Amp^r^ | Our lab |
| pBL | LacZ expression vector | This work |
| pBNA | Nattokinase expression vector | This work |

**Table S3** Primers

| Primer ID | Description | Sequence 5’-3’ | |  |
| --- | --- | --- | --- | --- |
| Primers used for construction integration plasmids | | | |  |
| *xpf*-US | Cloning *xpf* upstream flanking  fragment | GCGAAGCTTCCGTGTCATTATTGTGG | |  |
| *xpf*-UA | Cloning *xpf* upstream flanking fragment | CGGCTGCAGGTTTGAGCGTGCGTTT | |  |
| *xpf-*DS | Cloning *xpf* downstream flanking fragment | GCGCTGCAGTGCTGGAGGTGGCGGTGAT | |  |
| *xpf-*DA | Cloning *xpf* downstream flanking fragment | GCCGAATTCGCGCCTCTTTCCATTTGTCTTG | |  |
| *skfA*-US | Cloning *skfA* upstream flanking fragment | CCGGAAGCTTATTAGGCATCAGAA | |  |
| *skfA*-UA | Cloning *skfA* upstream flanking fragment | CGCCTGCAGCATAAGTAAACCTCC | |  |
| *skfA*-DS | Cloning *skfA* downstream flanking fragment | GCGCTGCAGGAATAGGGAGTTGAG | |  |
| *skfA*-DA | Cloning *skfA* downstream flanking fragment | GGCGGAATTCGTGTAAAGGCTGAT | |  |
| *lytC*-US | Cloning *lytC* upstream flanking fragment | CGGGAAGCTTTGGAGCGACTGTATT | |  |
| *lytC*-UA | Cloning *lytC* upstream flanking fragment | GGCCTGCAGCAGTGCCGTATCTATT | |  |
| *lytC*-DS | Cloning *lytC* downstream flanking fragment | CGGCTGCAGAGCAAGCGGTTTAT | |  |
| *lytC*-DA | Cloning *lytC* downstream flanking fragment | GCCGAATTCCCTATGCCAGTCAGTT | |  |
| *sdpC*-US | Cloning *sdpC* upstream flanking fragment | GGCGAAGCTTAAAAGAATAGGAGGTG | |  |
| *sdpC*-UA | Cloning *sdpC* upstream flanking fragment | GCGCTGCAGACGAGTTTAGGGT | |  |
| *sdpC*-DS | Cloning *sdpC* downstream flanking fragment | GCGCTGCAGATACGTTGCCAAAT | |  |
| *sdpC*-DA | Cloning *sdpC* downstream flanking fragment | GGGAATTCAGGGCTTGGTGTTGG | |  |
| *yGlA-*US | Cloning *yqxG-yqxH-cwlA* upstream flanking fragment | GCGGAAGCTTAAAACTGGCTCGTA | |  |
| *yGlA*-UA | Cloning *yqxG-yqxH- cwlA*  upstream flanking fragment | CCCCTGCAGTCTGTATGGCTTGG | |  |
| *yGlA*-DS | Cloning *yqxG-yqxH- cwlA*  downstream flanking fragment | GCCCTGCAGACGAAAGCGAAAC | |  |
| *yGlA*-DA | Cloning *yqxG-yqxH- cwlA*  downstream flanking fragment | CCCGAATTCCTGCTGTGCCTGT | |  |
| Primers used for identification of mutants | | |  | |
| *xpf-*seq S | Identification of *xpf* mutation | CCCATTCAAAGGAGCGTAT |  | |
| *xpf*-seq A | Identification of *xpf* mutation | GGCTGCCCGTCACAT |  | |
| *skfA*-seq S | Identification of *skfA* mutation | GCGTAACCCCTCTGGAT |  | |
| *skfA*-seq A | Identification of *skfA* mutation | GCGATAGCAAAACGAACA |  | |
| *lytC*-seq S | Identification of *lytC* mutation | GCCTTCTTGGTGGTCTTC |  | |
| *lytC*-seq A | Identification of *lytC* mutation | GGGCGTTCCTGCGTAT |  | |
| *sdpC*-seq S | Identification of *sdpC* mutation | CCGAGTTTTGGCTTGA |  | |
| *sdpC*-seq A | Identification of *sdpC* mutation | CGGGTTTAGGATACGAA |  | |
| *yGlA-*seq S | Identification of  *yqxG-yqxH-cwlA* mutation | GGCCGCACCTATACCCA |  | |
| *yGlA-*seq A | Identification of  *yqxG-yqxH-cwlA* mutation | GCATCCACCTAACTCATCG |  | |
| Primers to construct expression vectors | | | | |
| P43-SH | Cloning P43 fragment | ATCAAGCTTCATGCAGGCCGGG |  | |
| P43-AX | Cloning P43 fragment | TCCTCTAGAGTGTACATTCCTCTCTT |  | |
| lac-SX | Cloning *lacZ* fragment | GGTCTAGAATGACCATGATTACGGAT |  | |
| lacTT-AS | Cloning *lacZ* fragment, *trpA* terminator at the 5’ending and a *SphI* site between *lacZ* and *trpA* terminator | TCCCCCGGGAAAAAAGCCCGCTCATTAGGCGGGCTGCGCATGCTTATTTTTGACACCAG |  | |
| NA-SX | Cloning nattokinase fragment | GGTCTAGAGTGAGAAGCAAAAAATTGTG |  | |
| NA-AS | Cloning nattokinase fragment | ACATGCATGCTTATTGTGCAGCTGC |  | |

**Appendix S1 Genetic procedures for constructing plasmids and mutants**

All strains and plasmids used in this study are listed in Table **S1**, **S2**. Both *B. subtilis* and *E. coli* strains were propagated at 37°C in Luria-Burtani (LB) medium or on LB plates containing 1.5 % agar. LB medium for *E. coli* was supplemented with 100 µg/ml of ampicillin as selective pressure. Standard procedures were used for plasmid preparation, restriction enzyme digestions, ligations, transformations, and agarose gel electrophoresis (Maniatis et al., 1982). *B. subtilis* 168 was transformed by using the natural competence method (Anagnostopoulos and Spizizen, 1961). Spizizen minimal media was supplemented with glucose (0.5%), casein amino acid (0.02%) and amino acids. 50 µg/ml of Uracil nucleotide, 50 µg/ml of isoleucine and 20 µg/ml of tryptophan were added whenever required. When a plasmid containing the erythromycin resistance gene (*ermC*) was used for transformation in *B. subtilis*, erythromycin resistance was induced by adding 0.2µg/ml of erythromycin and incubating for 30 min at 37°C prior to plating. LB plates supplemented with 1µg/ml of erythromycin were used for *B. subtilis* transformation.

The *E. coli*/*B. subtilis* shuttle vector pNNB194 has an *E. coli* origin of replication, a temperature-sensitive *B. subtilis* origin of replication, an ampicillin resistance gene (*bla*) for selection in *E. coli*, and an erythromycin resistance gene (*ermC*) for selection in *B. subtilis.* Each gene deletion construct contained approximately 500bp to 800bp of DNA homologous to the flanking region of the targeted deletion sequence. They were achieved by PCR amplifying 200bp to 500bp at the upstream and downstream of each gene to be deleted respectively from *B.subtilis* 168. The primers used for each PCR are described in Table **S3.** The upstream *Hind*III/*Pst*I fragment and the downstream *Pst*I/*EcoR*I fragment were joined together into the *Hind*III/*EcoR*I site of pNNB194 to create the knockout vector.

For the strains listed in Table **S1**, gene deletion mutation was introduced into the chromosome through double-crossover homologous recombination events. The corresponding plasmid was introduced into *B. subtilis* competent cells by selecting for erythromycin resistance on LB plates at the permissive temperature of 30°C, and then it was carried out using the method of Connelly (Connelly, M. B. et al., 2004). Chromosomal integrations and deletions were conﬁrmed by using erythromycin markers and PCR analysis (primers are shown in Table **S3**).

The *E. coli*/*B. subtilis* shuttle vector pBE2 has an *E. coli* origin of replication from pBR322 and a *B. subtilis* origin of replication from pUB110, an ampicillin resistance gene (*bla*) for selection in *E. coli*, and a kanamycin resistance (*kan*) gene for selection in *B. Subtilis (*Guo X et al., 1991). There was a Sp6 promoter upstream the multiple cloning sites (MCS), but we found it was not so efficient. The constitutive promoter P43 (Wang, PZ et al., 1984) and *trpA* terminator (Kaltwasser, M. et al., 2002) from *B.subtilis* were employed to enhance the expression strength. The P43 promoter was amplified by PCR from the chromosomal DNA of *B.subtilis* 168 using primers P43-SH (containing *Hind*III site) and P43-AX (containing *XbaI* site). The *trpA* terminator was designed into the 5’ ending of the downstream primer lacTT-AS (containing *Sma*I site) of *lacZ*, and a *Sph*I site was designed between *lacZ* and *trpA* terminator for further manipulation. The *Xba*I/*Sma*I fragment coding lacZ was amplified by PCR from *E.coli* MG1655 using primers lac-SX (containing *XbaI* site) and lacTT-AS (containing *Sph*I site). Then the *Hind*III/*Xba*I fragment coding P43 and *Xba*I/*Sma*I fragment coding lacZ were joined together into pBE2 *Hind*III/*Sma*I site to generate pBL (Figure S1). The plasmid pBL contains P43 promoter upstream *Xba*I and *trpA* terminator downstream *Sph*I. The *Xba*I/*Sph*I fragment coding nattokinase was amplified by PCR from *B.natto* BN13 using primers NA-SX (containing *Xba*I site) and NA-AS (containing *Sph*I site), and then cloned into the *Xba*I/*Sph*I site of pBL to generate pBNA.

**Reference**

Connelly, M. B., Young, G. M., & Sloma, A. (2004). **Extracellular proteolytic activity plays a central role in swarming motility in *Bacillus subtilis*.** *Journal of bacteriology*, **186**(13), 4159-4167.

Kaltwasser, M, Wiegert, T & Schumann, W: **Construction and application of epitope-and green fluorescent protein-tagging integration vectors for *Bacillus subtilis*.** *Applied and environmental microbiology* 2002, **68**(5), 2624-2628.

Guo X, Xiong Z, Jia S & Xu Y: **The construction of multifunctional shuttle vectors of *Bacillus subtilis*- *Escherichia coli*.** *Chinese J Biotech* 1991. **7**: 224-229.

Wang, PZ, & Doi, RH: **Overlapping promoters transcribed by *Bacillus subtilis* sigma 55 and sigma 37 RNA polymerase holoenzymes during growth and stationary phases.** *Journal of Biological Chemistry* 1984, **259**(13), 8619-8625.

**Figure S1 Construction of expression plasmid**


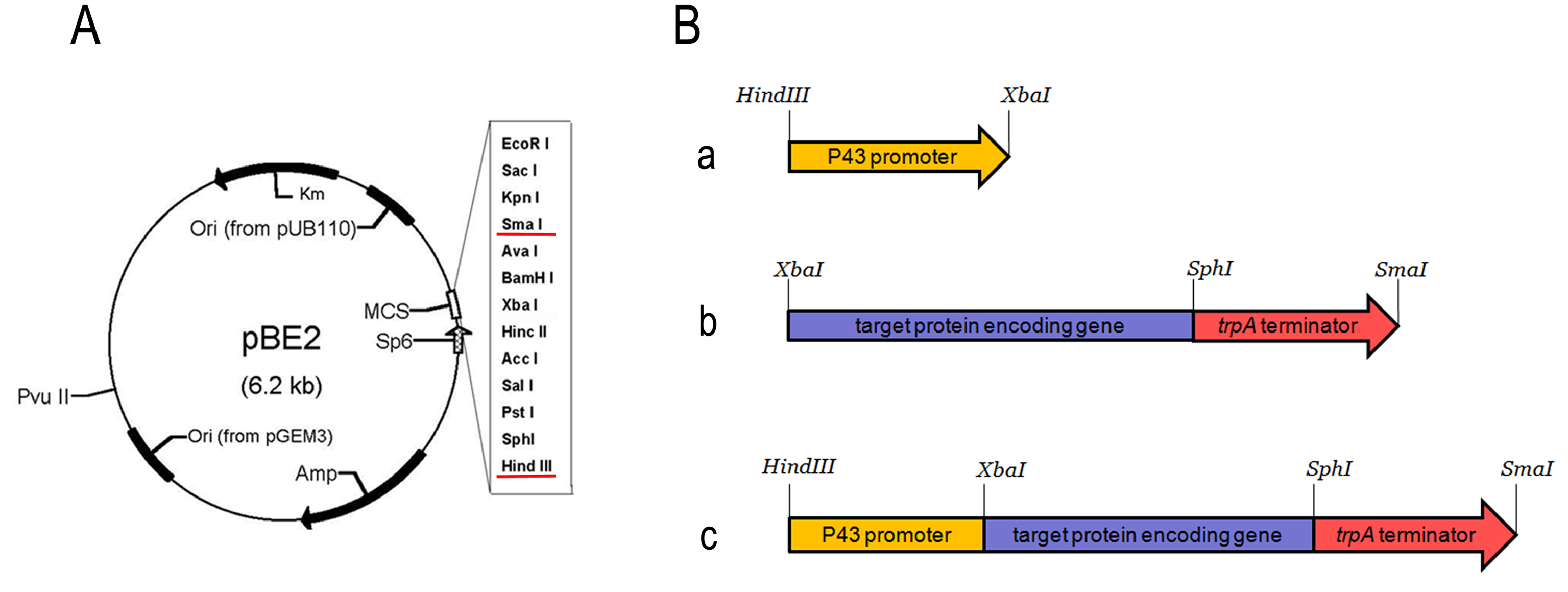


Fig.S1 Construction of expression plasmid

A. Vector pBE2 map. B. Construction of expression cassette P43-target gene-*trpA.* Fragment a: P43 promoter of *B.subtilis*; Fragment b: *trpA* terminator was placed into the downstream primer of *lacZ*, and there is a *SphI* site between *lacZ* and *trpA* termiator for further manipulation; Fragment c: the expression cassette P43-target gene-*trpA.* The fragment c with lacZ was cloned into pBE2 *Hind*III/*Sma*I site to generate pBL， and then applying *Xba*I/*Sph*I site *lacZ* was replaced by nattokinase coding gene *aprN* to generate the recombinant plasmid pBNA.

**Figure S2 Characteristic morphologies of the single mutants**


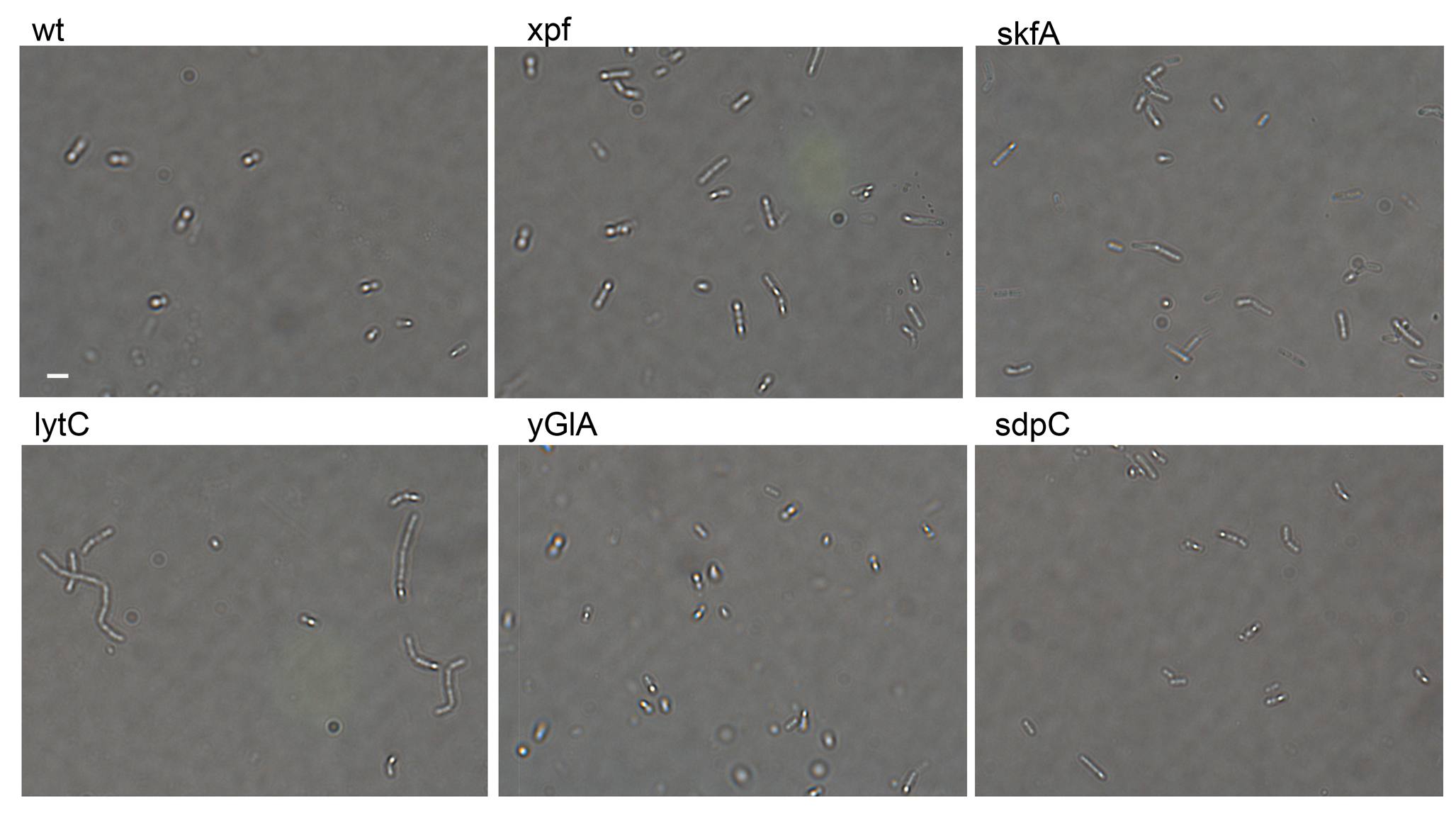


Fig.S2 Characteristic morphologies of the single mutants

After cultured in LB medium at 37℃ for 12h, 2μl of *B.subtilis* culture were followed by smear microscopy (bar, 2μm, Olympus IX51).

**Figure S3 Biomass yields of double lytic-gene deletion mutants.**


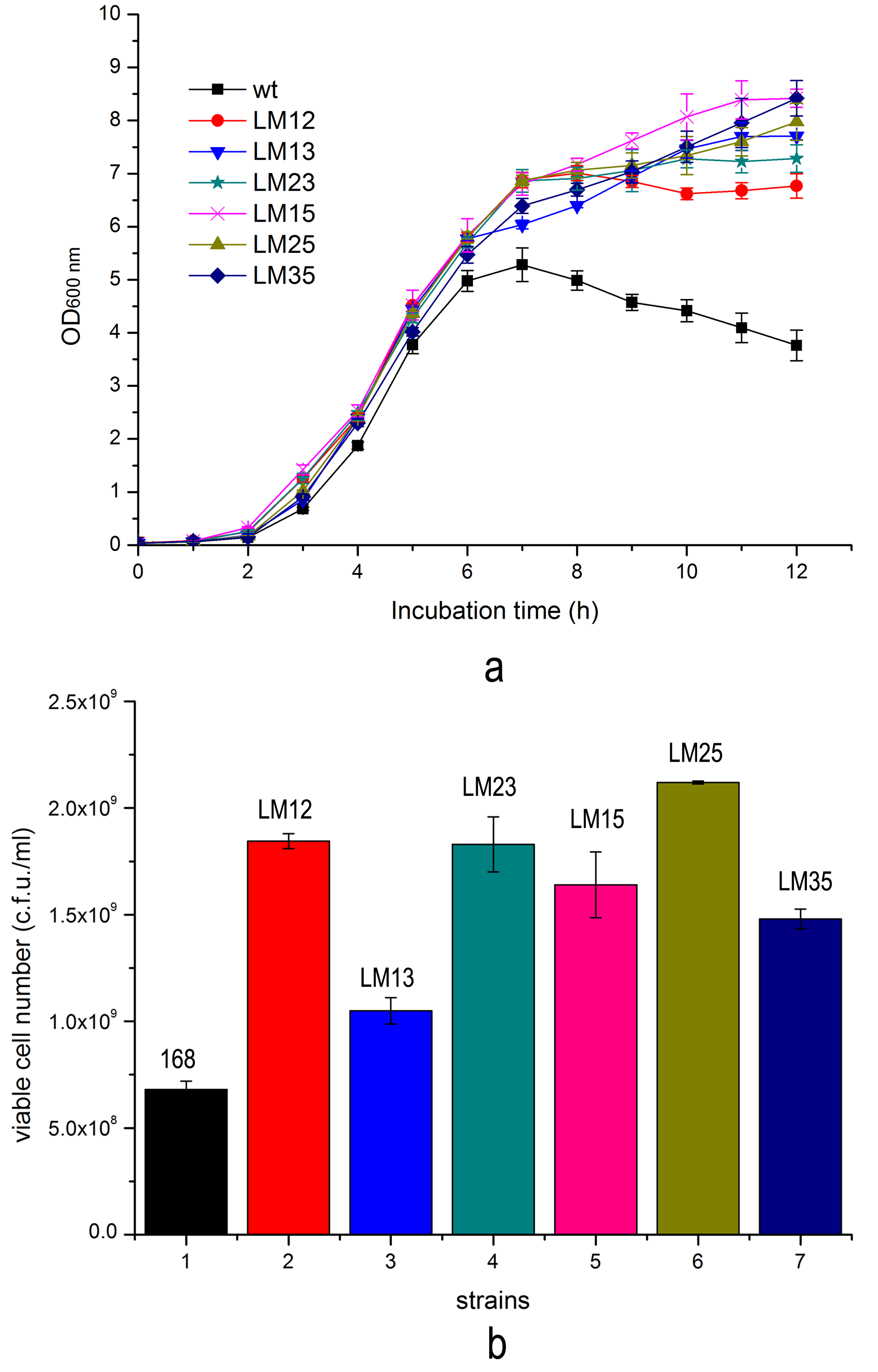


Fig.S3 Biomass yields of double lytic-gene deletion mutants. (a) OD_600_ of cultures of wild-type and double-deletion mutants: ■ wild-type ● LM12, ▼ LM13, ★ LM23, × LM15, ▲ LM25, ◆ LM35. (b) 12 h viable cell numbers: 1 Wild-type, 2 LM12, 3 LM13, 4 LM23, 5 LM15, 6 LM25, 7 LM35. The data represent the mean and SD from triplicate measurements of three different colonies of the same genotype for each mutant.

**Figure S4 Characteristic morphologies of the multiple mutants**


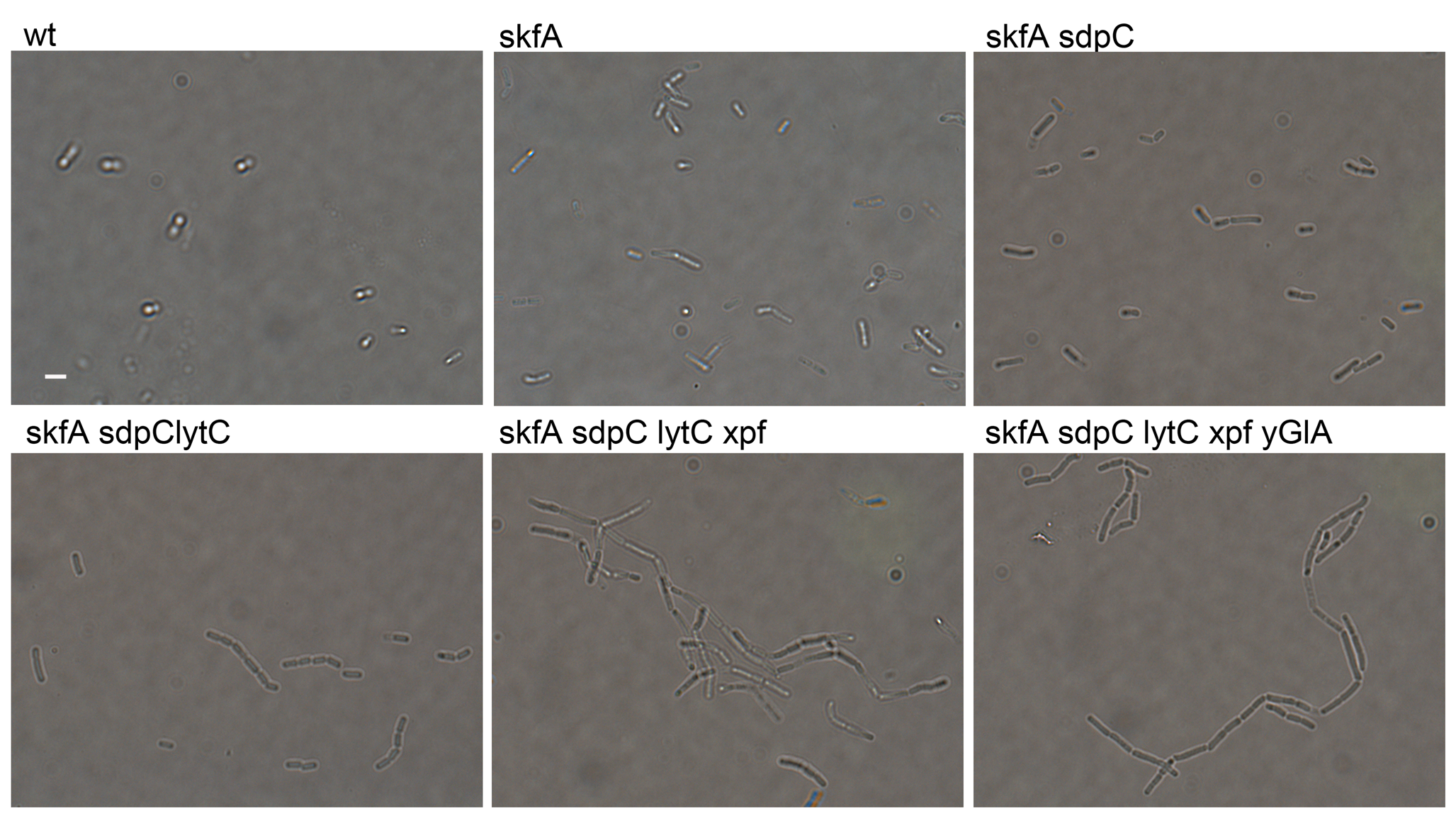


Fig.S4 Characteristic morphologies of the multiple mutants

After cultured in LB medium at 37℃ for 12h, 2μl of *B.subtilis* culture were followed by smear microscopy (bar, 2μm, Olympus IX51).

**Figure S5 RT-PCR analysis of *skfA* and *sdpC* transcription in *B.subtilis***

*
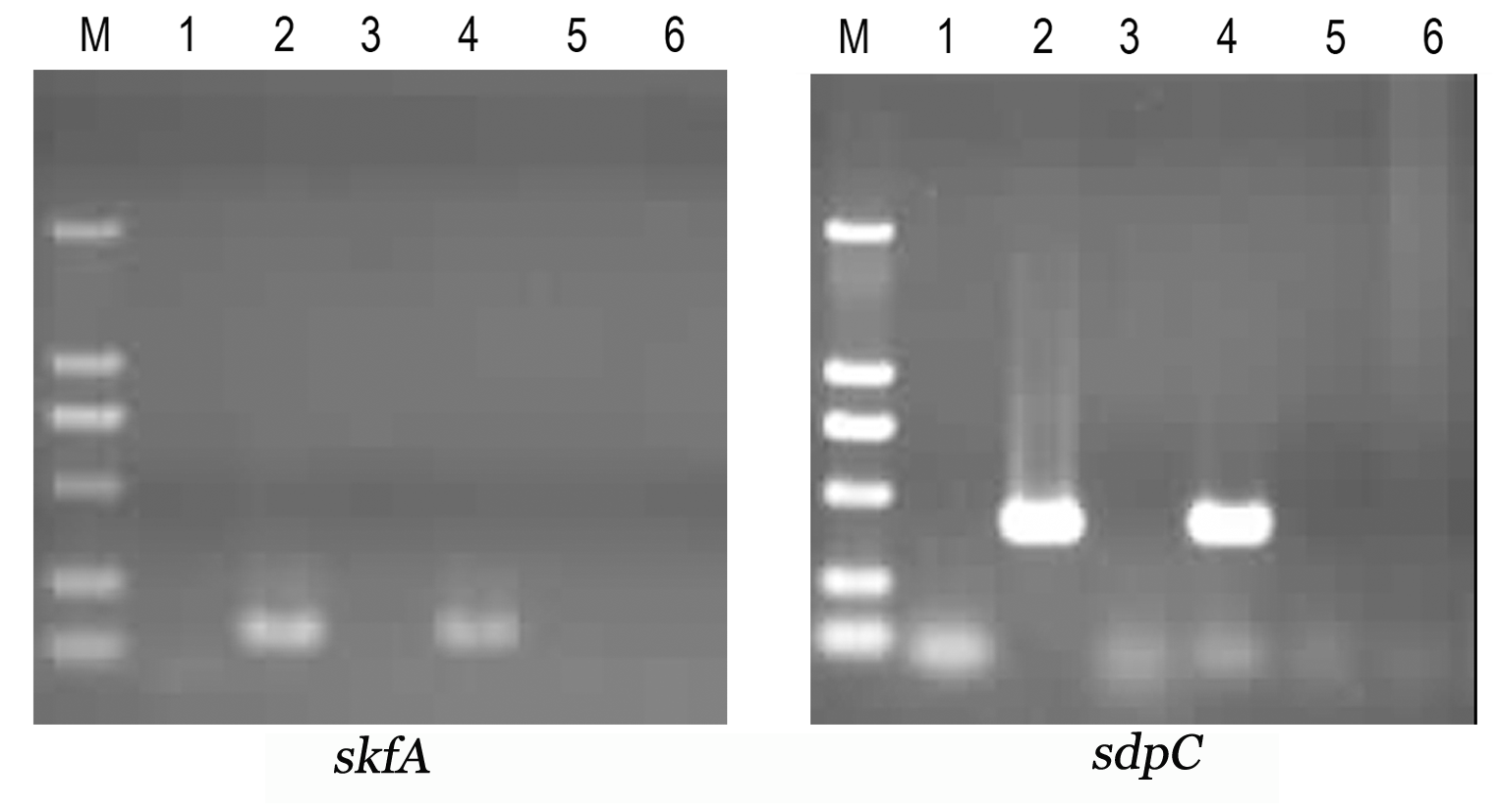
*

Fig.S5 RT-PCR analysis of *skfA* and *sdpC* transcription *in B.subtilis*

RNA of 6h *B.subtilis* cultures was extracted. After treated by genome DNA eraser and reverse transcribed into cDNA using random primers (Takara), PCR was performed using primers specific to *skfA* or *sdpC*. DNA marker DL2000 (M); negative control (1); *B.subtilis* 168 genome DNA as template (2); RNA of 6h *B.subtilis* 168 treated by genome DNA eraser as template (3); cDNA of 6h *B.subtilis* 168 as template (4); RNA of 6h mutant (LM2 or LM5) treated by genome DNA eraser as template (5); cDNA of 6h mutant(LM2 or LM5) as template (6).

SkfA and SdpC are sibling killing factors implicated in sporulation, but from the result of their RT-PCR, we can see that they were transcripted even at 6h.

**Figure S6 Spore production by the mutant strains**


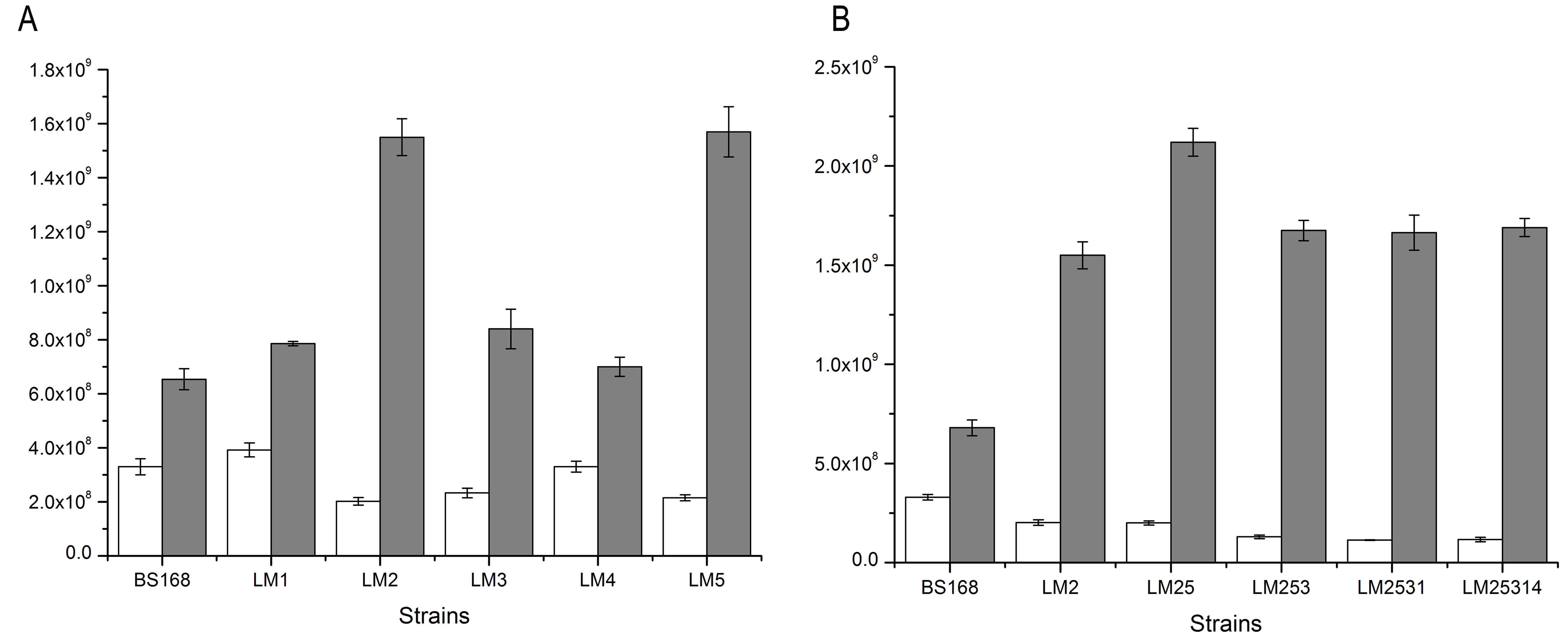


Figure S6 Spore production by the mutant strains

A: spore production by the single-deletion mutants: *B. subtilis* 168, LM1, LM2, LM3, LM4, LM5 □ spore numbers, ■ viable cell numbers. B: spore production by the multiple-deletion mutants: *B. subtilis* 168, LM2, LM25, LM253, LM2531, LM25314. □ spore numbers, ■ viable cell numbers. Strains were cultured at 37ºC for 12 h.

**Figure S7 Standard curve of nattokinase**


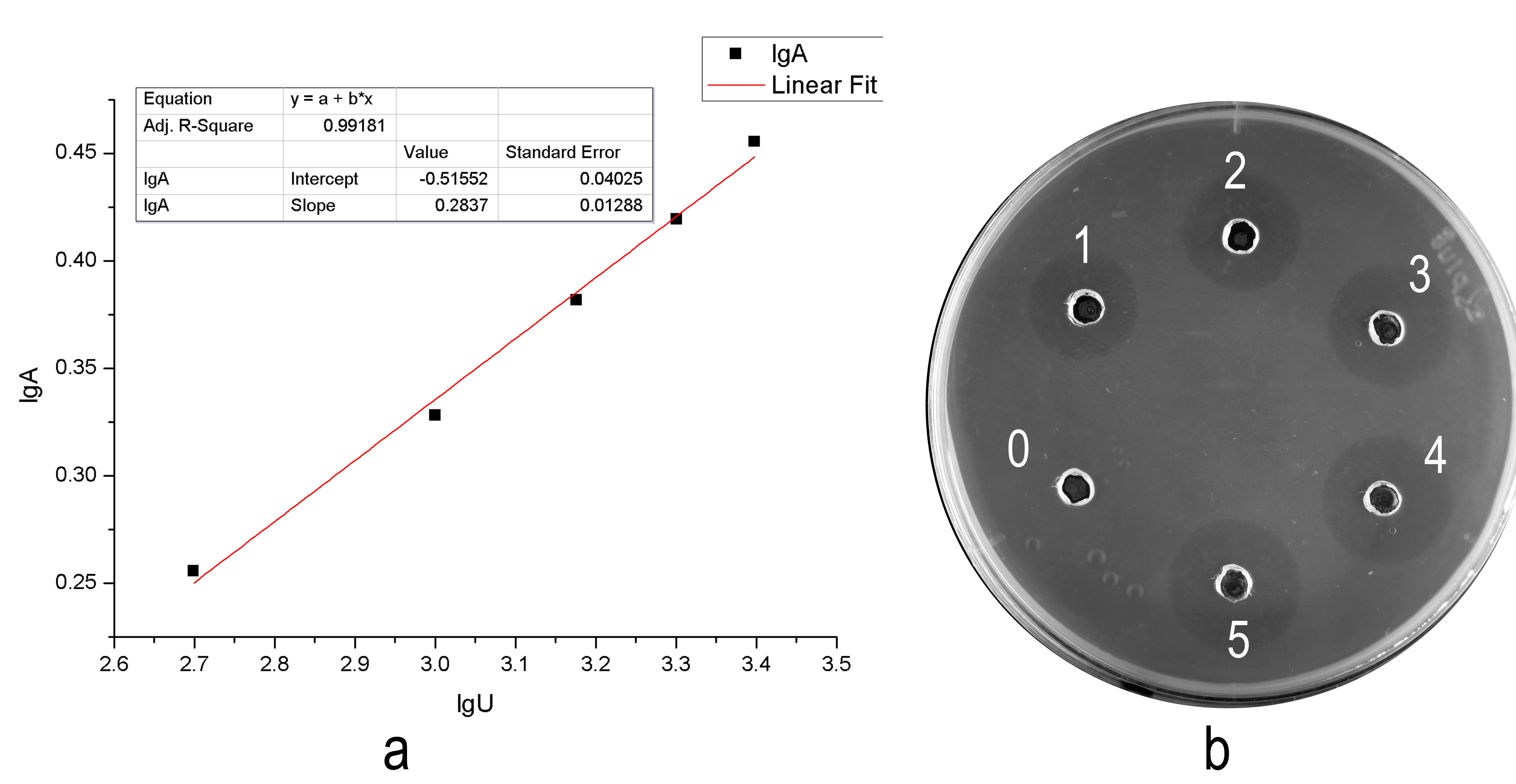


Fig.S7 Standard curve of nattokinase

Fibrinolytic activity assay was carried out using urokinase as standard according to the method of Astrup (Astrup, T., & Müllertz, S., 1952). As reported the lysis clear zone area A and the enzyme activity U correspond to the formula A= k*U^a^ or lgA=a*lgU+b. Five concentrations 500IU/ml, 1000IU/ml, 1500IU/ml, 2000IU/ml and 2500IU/ml were used, they corresponded to 1-5 in fig b respectively, and 0 was the control using dilution buffer. Fig a in the left was the standard curve we got, lgA= 0.2837 lgU - 0.5155, in which R^2^ = 0.9918. Using this formula we can figure out the activity of our samples.

**Reference**

Astrup, T., & Müllertz, S. (1952). **The fibrin plate method for estimating fibrinolytic activity**. *Archives of biochemistry and biophysics*, **40**(2), 346-351.
